# Characterisation of homologues of known and putative dynein assembly factors in a *Drosophila *model

**DOI:** 10.1186/2046-2530-4-S1-P80

**Published:** 2015-07-13

**Authors:** P zur Lage, D Moore, G Mali, E Hall, P Mill, A Jarman

**Affiliations:** 1 SBMS/CIP, University of Edinburgh, Edinburgh, United Kingdom; 2 MRC IGMM, University of Edinburgh, Edinburgh, United Kingdom

## 

With only two types of ciliated cells, *Drosophila *is a useful organism in which to study conserved aspects of ciliogenesis. Cilia with motile characteristics in *Drosophila *are represented just by the sperm flagella and the sensory receivers of chordotonal neurons, which are proprioceptive and auditory sensory neurons.

We recently used *Drosophila *to identify two new putative dynein arm assembly factors, CG11253 (ZMYND10 homologue) and CG31320 (HEATR2 homologue): impairment of function of either gene results in flies with immotile sperm and defective sensory transduction due to lack of dynein arms in the cilia. We have sought to extend these observations to other known or putative dynein assembly factor homologues to determine how much of this pathway is conserved and set up *Drosophila *as a model for exploring the function of these proteins in further detail. This analysis has used transcriptomic data of developing chordotonal neurons, gene regulatory analysis (regulation by Rfx and Fox factor, Fd3F), genetic analysis of sensation and male fertility, and protein interaction analysis. Currently, we are also carrying out mass spectroscopy.

